# Nomogram to predict primary non-response to infliximab in patients with Crohn’s disease: a multicenter study

**DOI:** 10.1093/gastro/goaa069

**Published:** 2020-11-12

**Authors:** Xiao-Qi Ye, Jing Cai, Qiao Yu, Xiao-Cang Cao, Yan Chen, Mei-Xin Rao, Bai-Li Chen, Yao He, Zhi-Rong Zeng, Hao Chen, Yi-Mou Lin, Qian Cao, Min-Hu Chen, Sheng-Hong Zhang

**Affiliations:** 1 Department of Gastroenterology, The First Affiliated Hospital, Sun Yat-sen University, Guangzhou, Guangdong, P. R. China; 2 Department of Gastroenterology, Sir Run Run Shaw Hospital, Zhejiang University School of Medicine, Hangzhou, Zhejiang, P. R. China; 3 Department of Gastroenterology, Wenzhou Central Hospital, Wenzhou, Zhejiang, P. R. China; 4 Department of Gastroenterology, the Second Affiliated Hospital, Zhejiang University School of Medicine, Hangzhou, Zhejiang, P. R. China; 5 Department of Gastroenterology, Tianjin Medical University General Hospital, Tianjin, P. R. China

**Keywords:** Crohn’s disease, infliximab, nomogram, primary non-response, mucosal healing

## Abstract

**Background:**

Infliximab (IFX) is effective at inducing and maintaining clinical remission and mucosal healing in patients with Crohn’s disease (CD); however, 9%–40% of patients do not respond to primary IFX treatment. This study aimed to construct and validate nomograms to predict IFX response in CD patients.

**Methods:**

A total of 343 patients diagnosed with CD who had received IFX induction from four tertiary centers between September 2008 and September 2019 were enrolled in this study and randomly classified into a training cohort (*n* = 240) and a validation cohort (*n* = 103). The primary outcome was primary non-response (PNR) and the secondary outcome was mucosal healing (MH). Nomograms were constructed from the training cohort using multivariate logistic regression. Performance of nomograms was evaluated by area under the receiver-operating characteristic curve (AUC) and calibration curve. The clinical usefulness of nomograms was evaluated by decision-curve analysis.

**Results:**

The nomogram for PNR was developed based on four independent predictors: age, C-reactive protein (CRP) at week 2, body mass index, and non-stricturing, non-penetrating behavior (B1). AUC was 0.77 in the training cohort and 0.76 in the validation cohort. The nomogram for MH included four independent factors: baseline Crohn’s Disease Endoscopic Index of Severity, CRP at week 2, B1, and disease duration. AUC was 0.79 and 0.72 in the training and validation cohorts, respectively. The two nomograms showed good calibration in both cohorts and were superior to single factors and an existing matrix model. The decision curve indicated the clinical usefulness of the PNR nomogram.

**Conclusions:**

We established and validated nomograms for the prediction of PNR to IFX and MH in CD patients. This graphical tool is easy to use and will assist physicians in therapeutic decision-making.

## Introduction

Crohn’s disease (CD) is a chronic and refractory inflammatory bowel disease that can affect any portion of the gastrointestinal tract from the mouth to the perianal area. CD is mainly characterized by prolonged diarrhea with crampy abdominal pain, weight loss, and fever, with or without gross bleeding [1]. A substantial portion of patients with CD may develop a series of complications, such as fistula, abdominal abscess, and bowel obstruction [2–4], which decrease quality of life, cause physical and psychological morbidity, and increase mortality [5–7]. CD has been reported to be associated with significantly increased healthcare and economic burdens [8]. As the incidence of CD is increasing rapidly in Asian countries, especially China [9], efficient and cost-effective therapies are urgently being sought.

Infliximab (IFX), a chimeric anti-human necrosis factor-α monoclonal antibody, is the most cost-effective biologic for CD patients who fail to respond to standard therapy [10]. It is also well known that IFX is effective for inducing and maintaining remission in CD patients [11]. Nowadays, the therapeutic goal for CD patients has evolved from remission to mucosal healing (MH)—a more ambitious goal. As a ‘treat-to-target’ strategy, MH decreases the risks of surgery, new penetrating events, and new stenosis, thus reducing disability caused by CD [12].

However, 9%–40% of CD patients exhibit primary non-response (PNR) to IFX [13]. There is no consensus on the definition of PNR; however, PNR features little improvement after initiating the induction therapy of IFX. Since IFX is expensive, especially for patients in Asia, where the majority of the populace are not covered by insurance [14], precision medicine has been suggested to optimize treatment strategies. Furthermore, IFX is related to several adverse events, such as infusion reactions, serious infection, and malignancy [15–17], and treating non-responders with IFX increases exposure to adverse events and delays initiation of other effective CD treatments.

Several risk factors associated with PNR have been explored, such as age, sex, body mass index (BMI), smoking, disease duration, small-bowel involvement, stenosing and/or penetrating phenotype, FAS-L, and caspase-9 in apoptosis-related genes [11, 13]. The effects of these risk factors can be partly explained by pharmacokinetics, pharmacodynamics, and pharmacogenetics of monoclonal antibodies [11, 18]. However, the exact mechanism of PNR to IFX has not been elucidated.

By combining these risk factors, Billiet *et al.* [19] constructed a matrix model that makes predictions based on age, BMI, and prior surgery history, but the model was not validated in external or multicenter cohorts. Tang *et al.* [20] and Jung *et al.* [21] developed effective prediction models by combining clinical data and genetic factors; however, since genetic analyses are not currently applied in routine examinations, these models are not practical.

Thus, we aimed to use routine clinical data to construct and validate nomograms of IFX response in CD patients from a multicenter cohort; these nomograms could aid in therapeutic decision-making.

## Patients and methods

### Study population

We collected data of patients with CD treated in four inflammatory bowel disease (IBD) centers in China between September 2008 and September 2019, including the First Affiliated Hospital of Sun Yat-sen University (Guangzhou, China), Sir Run Run Shaw Hospital of Zhejiang University School of Medicine (Hangzhou, China), the Second Affiliated Hospital of Zhejiang University School of Medicine (Hangzhou, China), and the General Hospital of Tianjin Medical University (Tianjin, China). Inclusion criteria were as follows: (i) a diagnosis of CD based on clinical, endoscopic, radiographic, and histological evidence in the center; (ii) endoscopic active with a Crohn’s Disease Endoscopic Index of Severity (CDEIS) of > 3 before IFX treatment; and (iii) complement of induction of IFX of 5 mg/kg at weeks 0, 2, and 6 in the center. Exclusion criteria included (i) previous ileac, colonic, or ileocolonic resection; (ii) lack of evaluation of endoscopy before induction of IFX or at week 14 after the first induction of IFX; and (iii) incomplete data. Patients with previous ileum, colon, or ileocolic resection were excluded because the standard evaluation of post-surgical recurrence is Rutgeerts score, rather than CDEIS. We decided that 70% of enrolled patients were used to derive the model and 30% of them were used for the validation study. Since patients were recruited from various hospitals, the randomization was supposed to be stratified by four centers.

### Data collection

A predetermined data sheet was used to collect information including age, sex, weight, height, smoking habits, surgical history, disease duration, disease localization, disease behavior (as defined by the Montreal Classification [22]), presence of extra-intestinal manifestations, concomitant therapy, C-reactive protein (CRP), serum albumin concentration, and CDEIS at the initiation of IFX treatment. Follow-up data included CRP at every IFX infusion and CDEIS at week 14. CDEIS was calculated by experienced IBD clinicians.

### Outcomes and definitions

The primary outcome was PNR, which was defined as a decrease in CDEIS of < 50% from baseline at week 14 after the first IFX infusion. On the contrary, response was defined as a decrease in CDEIS of ≥ 50% from baseline at week 14. The secondary outcome was MH, defined as CDEIS < 1.5 at week 14. Prior surgery was defined as resection of a part of the gut, strictureplasty for stenosing complications, or a fistulectomy/fistulotomy for complicated perianal disease [19].

### Construction of nomograms

First, Spearman’s correlation analyses were performed to detect multicollinearity. Correlation factors >0.7 were considered significant and collinear factors should be excluded from analysis to decrease bias. Logistic regression was used to select risk factors for univariate analysis. Significant variables were included in a multivariate logistic-regression analysis. The final model in multivariate regression was selected by backward step-down analysis based on Akaike’s Information Criterion. A nomogram was developed based on the multivariate logistic-regression model. A nomogram is an intuitive and quantitative tool to predict the probability of outcomes.

### Assessment of nomogram performance

Discriminative ability was assessed by the area under the receiver-operating characteristic curve (AUC). The values of AUC are between 0.5 and 1.0, with 0.5 corresponding to a model with no discriminatory ability and 1.0 corresponding to perfect discrimination. The comparison of AUCs was conducted by Delong’s test for single factors and 2,000 bootstrap resamples for the matrix model [19]. Calibration was tested by a Hosmer-Lemeshow goodness-of-fit test after splitting the sample into quintiles. This test assessed how well the model fits observed data, with *P* > 0.05 indicating no evidence of poor fit. Calibration curves were presented to depict the relationships between predicted probabilities and observed frequencies. The overlap with the reference line indicates perfect agreement on the model.

### Clinical utility of nomograms

Decision-curve analysis (DCA) was conducted to calculate the net benefits at different threshold probabilities in the combined training and validation cohorts. The optimal cut-off value was selected by maximizing the sum of the sensitivity and specificity on the Youden index from the training group.

### Ethics statement

This study was approved by the Research Ethics Committee of the First Affiliated Hospital of Sun Yat-sen University, Sir Run Run Shaw Hospital of Zhejiang University School of Medicine, the Second Affiliated Hospital of Zhejiang University School of Medicine, and the General Hospital of Tianjin Medical University (No. [2019] 349). Written informed consent was obtained from all patients in accordance with ethical guidelines of the Declaration of Helsinki. The study protocol was registered online at http://www.chictr.org.cn with the registration number ChiCTR1900026091.

### Statistical analysis

Continuous variables were presented as medians and interquartile range (IQR), and were compared using Wilcoxon rank-sum tests. Categorical variables were presented as counts and percentages of the cohort, and were compared by using Chi-squared tests or Fisher’s exact tests, as appropriate. All statistical analyses were performed in R software (version 3.6.1). Randomization was conducted using the ‘caTools’ package. Receiver-operating characteristic (ROC) curves were plotted using the ‘pROC’ package. Nomograms and calibration curves were performed using the ‘rms’ package. The Hosmer-Lemeshow test was analysed using the ‘ResourceSelection’ package. DCA was generated using the ‘rmda’ package. *P* values < 0.05 were considered statistically significant.

## Results

### Patient characteristics

A total of 343 patients diagnosed with CD who had received IFX induction were enrolled in this study. After applying randomization stratified by centers, these patients were randomly divided into a training cohort (*n* = 240) and a validation cohort (*n* = 103). The baseline characteristics of the two cohorts are displayed in Table 1. There was no difference in the PNR rate between the two cohorts (25.8% and 25.2% in the training and validation cohorts, respectively; *P* = 1.000). The MH rate was also not significantly different between the two cohorts (39.6% and 35.9%, respectively; *P* = 0.605). No significant differences between the two cohorts were found in any variables. No correlation factors were > 0.40 in Spearman’s correlation analyses.

**Table 1.** Baseline characteristics of 343 patients with Crohn’s disease

Characteristic	Training cohort (*n* = 240)	Validation cohort (*n* = 103)	*P* value
Age, years, median (IQR)	23 (18–31)	21 (18–27.5)	0.153
Males, *n* (%)	175 (72.9)	77 (74.8)	0.826
Body mass index, kg/m^2^, median (IQR)	18.3 (15.9–20.0)	18.4 (16.2–20.2)	0.896
Smoking, *n* (%)	12 (5.0)	5 (4.9)	1.000
Duration of disease, months, median (IQR)	12 (5–36)	12 (6–36)	0.668
Disease location, *n* (%)			0.181
L1: Ileal	19 (7.9)	4 (3.9)	
L2: Colonic	20 (8.3)	5 (4.8)	
L3: Ileocolonic	201 (83.8)	94 (91.3)	
Upper-tract involvement	60 (25.0)	28 (27.2)	0.772
Disease behavior, *n* (%)			0.525
B1: Non-stricturing, non-penetrating	169 (70.4)	79 (76.7)	
B2: Stricturing	61 (25.4)	21 (20.4)	
B3: Penetrating	10 (4.2)	3 (2.9)	
Perianal disease	148 (61.7)	67 (65.0)	0.637
Presence of extra-intestinal manifestations, *n* (%)	52 (21.7)	22 (21.4)	1.000
Prior surgery, *n* (%)	91 (37.9)	40 (38.8)	0.969
Baseline CRP, mg/L, median (IQR)	21.0 (9.6–41.9)	24.3 (10.2–41.7)	0.511
CRP ≥ 5 mg/L at week 2, *n* (%)	53 (22.1)	26 (25.2)	0.619
Baseline albumin, g/L, median (IQR)	35.0 (31.0–39.0)	34.4 (31.0–37.7)	0.194
Baseline CDEIS, median (IQR)	10 (7–13)	11 (7–14)	0.371
Concomitant therapy, *n* (%)			
Azathioprine or 6-mercaptopurine	116 (48.3)	54 (52.4)	0.563
Corticosteroids	21 (8.8)	9 (8.7)	1.000
Primary non-response, *n* (%)	62 (25.8)	26 (25.2)	1.000
Mucosal healing, *n* (%)	95 (39.6)	37 (35.9)	0.605

CDEIS, Crohn's Disease Endoscopic Index of Severity; CRP, C-reactive protein; IQR, interquartile range.

### Nomogram for PNR

#### Construction of the nomogram

**Figure 1.**
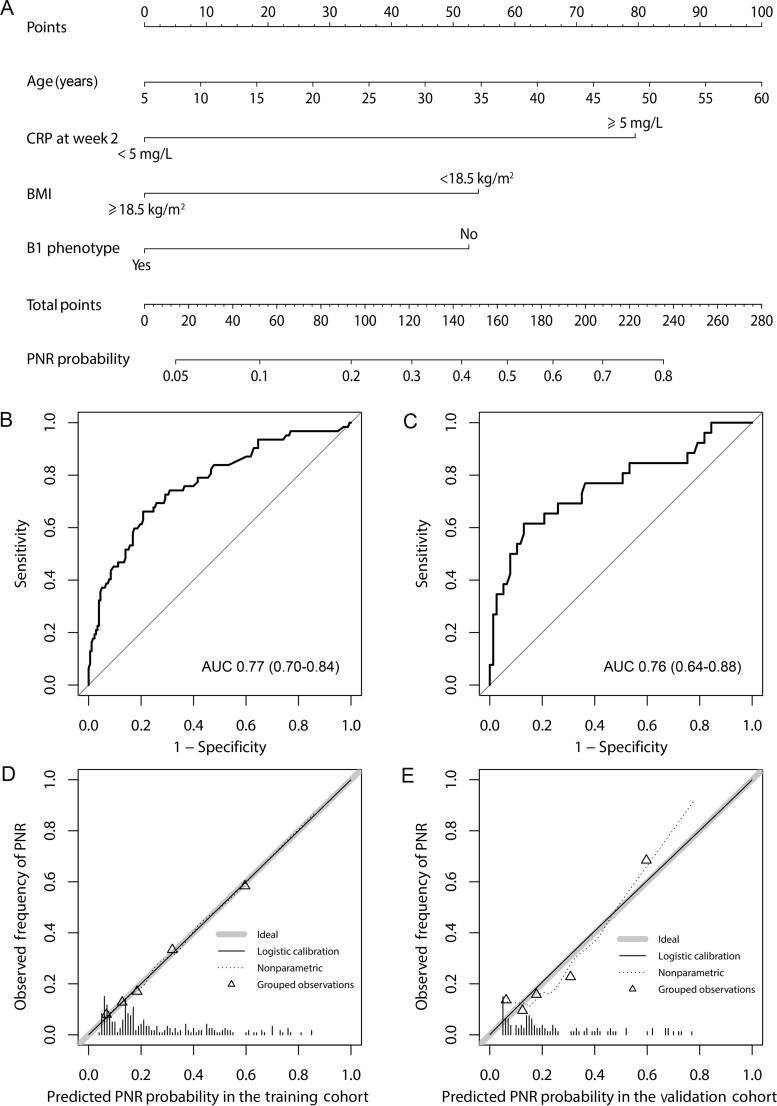
The display and evaluation of the model for primary non-response (PNR) to infliximab in patients with Crohn’s disease. (A) The nomogram to predict the probability of PNR. Receiver-operating characteristic (ROC) curves for the PNR nomogram in the training cohort (B) and validation cohort (C). Plots (D) and (E) show the calibration curves of the training and validation cohorts, respectively. The distribution of the predicted probabilities of PNR is shown at the bottom of the graphs. The triangles indicate the observed frequencies of PNR by the quintiles of the predicted probability. AUC, area under ROC curve; B1, non-stricturing, non-penetrating behavior; BMI, body mass index; CRP, C-reactive protein.

The results of univariate and multivariate analyses of PNR are listed in Table 2. In the training cohort, patients with PNR were older (*P* = 0.025) and had lower BMI (*P* = 0.001), less B1 phenotype (*P* < 0.001), higher CRP at baseline (*P* = 0.006) and the second week (*P* < 0.001), and lower serum albumin levels (*P* = 0.003) than those who responded to IFX. All the above variables with *P* <0.05 in the univariate analyses entered multivariate-regression analysis. Finally, age at first IFX [odds ratio (OR) 1.04; 95% confidence interval (CI), 1.00–1.07], BMI ≥18.5 kg/m^2^ (OR 0.39; 95% CI, 0.19–0.82), B1 phenotype (OR 0.36; 95% CI, 0.18–0.70), and CRP ≥ 5 mg/L at week 2 (OR 4.01; 95% CI, 1.92–8.40) were selected and further utilized to construct the nomogram (Figure 1A).

**Table 2.** Univariate and multivariate logistic analyses for primary non-response in 240 patients with Crohn’s disease of the training cohort

Variable	Univariate analysis		Multivariate analysis	
	*P* value	OR (95% CI)	*P* value	OR (95% CI)
Age (1-year increase)	0.025	1.03 (1.00–1.06)	0.033	1.04 (1.00–1. 07)
Male sex	0.945	0.98 (0.51–1.87)		
BMI ≥18.5 kg/m^2^	0.001	0.36 (0.19–0.67)	0.013	0.39 (0.19–0.82)
Smoking	0.545	1.47 (0.43–5.05)		
Duration of disease (1-month increase)	0.069	1.01 (1.00–1.01)		
Disease location				
L1: Ileal		Reference		
L2: Colonic	0.228	0.38 (0.08–1.82)		
L3: Ileocolonic	0.625	0.78 (0.28–2.15)		
Upper-tract involvement	0.865	1.06 (0.55–2.06)		
B1 phenotype	< 0.001	0.32 (0.17–0.59)	0.003	0.36 (0.18–0.70)
Perianal disease	0.200	0.68 (0.38–1.23)		
Presence of extra-intestinal manifestations	0.385	0.72 (0.34–1.51)		
Prior surgery	0.096	0.59 (0.32–1.10)		
Baseline CRP (1-mg/L increase)	0.006	1.01 (1.00–1.02)	0.368	1.00 (0.99–1.02)
CRP ≥5 mg/L at week 2	< 0.001	5.04 (2.62–9.70)	< 0.001	4.01 (1.92–8.40)
Baseline albumin (1-g/L increase)	0.003	0.92 (0.87–0.97)	0.447	0.97 (0.91–1.04)
Baseline CDEIS (1-unit increase)	0.259	1.03 (0.98–1.10)		
Concomitant therapy with AZA/6-MP	0.760	1.09 (0.61–1.95)		
Concomitant therapy with corticosteroids	0.764	1.16 (0.43–3.15)		

6-MP, 6-mercaptopurine; AZA, azathioprine; B1, non-stricturing, non-penetrating behavior; BMI, body mass index; CI, confidence interval; CDEIS, Crohn’s Disease Endoscopic Index of Severity; CRP, C-reactive protein; OR, odds ratio.

To use the nomogram, we drew a vertical line straight upward to the points axis for each predictor, added up the points from each predictor, and drew a vertical line downward from the total points axis to determine the probability of PNR. For example, a 20-year-old male was diagnosed with CD of B1 phenotype; his BMI was 16.5 kg/m^2^; CRP at week 2 after first IFX was 1.0 mg/L. We adopted the nomogram to this case: Age 20 = 27, CRP at week 2 lower than 5 mg/L = 0, BMI lower than 18.5 kg/m^2^ = 54, B1 phenotype = 0, total point = 81. The probability for PNR was 0.16.

#### Performance of the nomogram

The AUCs were 0.77 (95% CI: 0.70–0.84) and 0.76 (95% CI: 0.64–0.88) in the training and validation cohorts, respectively (Figure 1B and C). *P*-values of the Hosmer-Lemeshow goodness-of-fit test were >0.05 in both the training cohort and the validation cohort. Calibration curves showed excellent agreement between the nomogram prediction and actual PNR rate in the training and validation cohorts (Figure 1D and E).

#### Comparison of the nomogram with single factors and the matrix model

**Figure 2.**
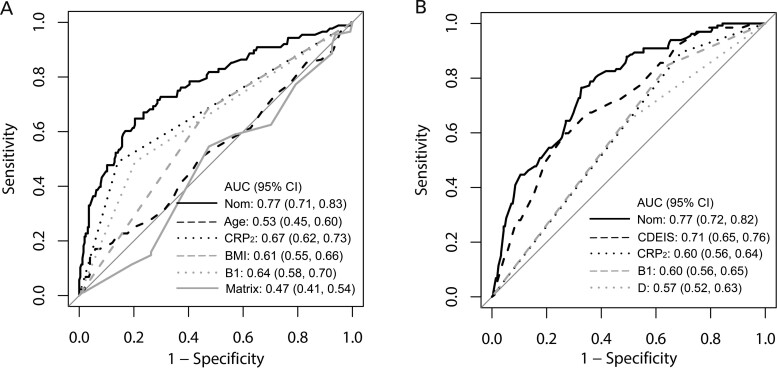
Receiver-operating characteristic (ROC) curves in all 343 patients. (A) Comparison of the primary non-response (PNR) nomogram with single factors and the matrix model. (B) Comparison between the mucosal healing (MH) nomogram and single factors. AUC, area under ROC curve; B1, non-stricturing, non-penetrating behavior; BMI, body mass index; CDEIS, Crohn's Disease Endoscopic Index of Severity; CRP_2_, C-reactive protein level at week 2; D, duration of disease; Matrix, the matrix model; Nom, nomogram.

As shown in Figure 2A, the AUC in the combined training and validation cohorts was 0.77, which had a significantly higher predictive accuracy for PNR than age at first IFX (AUC = 0.53, *P* < 0.001), CRP at week 2 (AUC = 0.67, *P* < 0.001), BMI (AUC = 0.61, *P* < 0.001), or B1 phenotype (AUC = 0.64, *P* < 0.001) alone. We also compared the discrimination of the nomogram with that of an existing matrix model [19]. The AUC of nomogram for PNR was significantly higher than that of the matrix model (AUC = 0.47, *P* < 0.001).

#### Clinical utility of the nomogram

**Figure 3.**
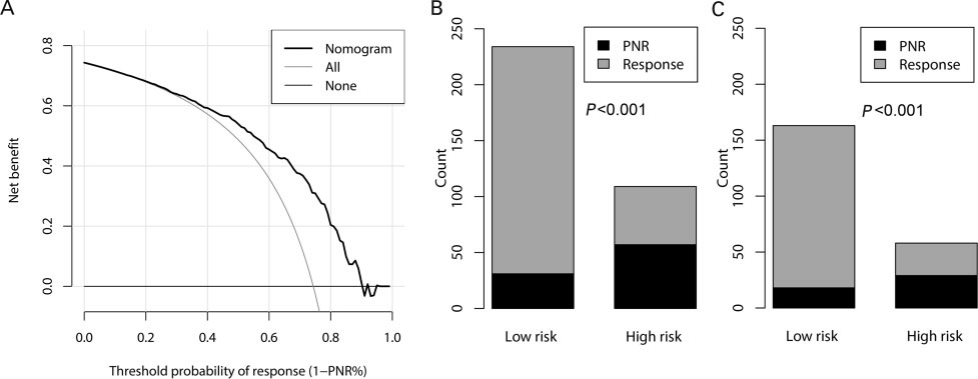
Evaluation of the clinical utility of the nomogram. (A) Decision-curve analysis for the primary non-response (PNR) nomogram. The *y*-axis represents net benefit and the *x*-axis shows the threshold probability of response. The ‘All’ line refers to the hypothesis that all patients were treated with infliximab and the ‘None’ line to the assumption that no patient was treated with infliximab. The probability of response is equal to 1 – probability of PNR (PNR%). The PNR nomogram is superior to the curve for ‘treat all’ or ‘treat none’ for thresholds of response between 0.15 and 0.90. Plots (B) and (C) show the risk-classification performance of the nomogram in overall patients and patients with perianal disease, respectively.

The DCA for the nomogram of PNR was plotted (Figure 3A). The net benefit was positive when the threshold probability for response, which is equal to 1 − probability of PNR, was within a range of 0.15–0.90. In other words, when the threshold probability of PNR was between 0.10 and 0.85, the nomogram added more net benefit than ‘treat-all’ or ‘treat-none’ strategies.

Based on the Youden index in the training cohort, the overall patients were divided into low- and high-risk groups by a cut-off value of 0.296 of PNR probability (equal to 121 of the total points in the nomogram). Patients with high risk had greater probability of PNR in overall patients (47.1% vs 14.3%, *P* < 0.001; Figure 3B). Since >60% of patients were complicated with perianal disease, we also evaluated the utility of the nomogram for these patients and found that the nomogram had good discriminatory ability for PNR in luminal disease for them (11.0% vs 50.0%, *P* < 0.001; Figure 3C).

### Nomogram for MH

#### Construction of the nomogram

**Figure 4.**
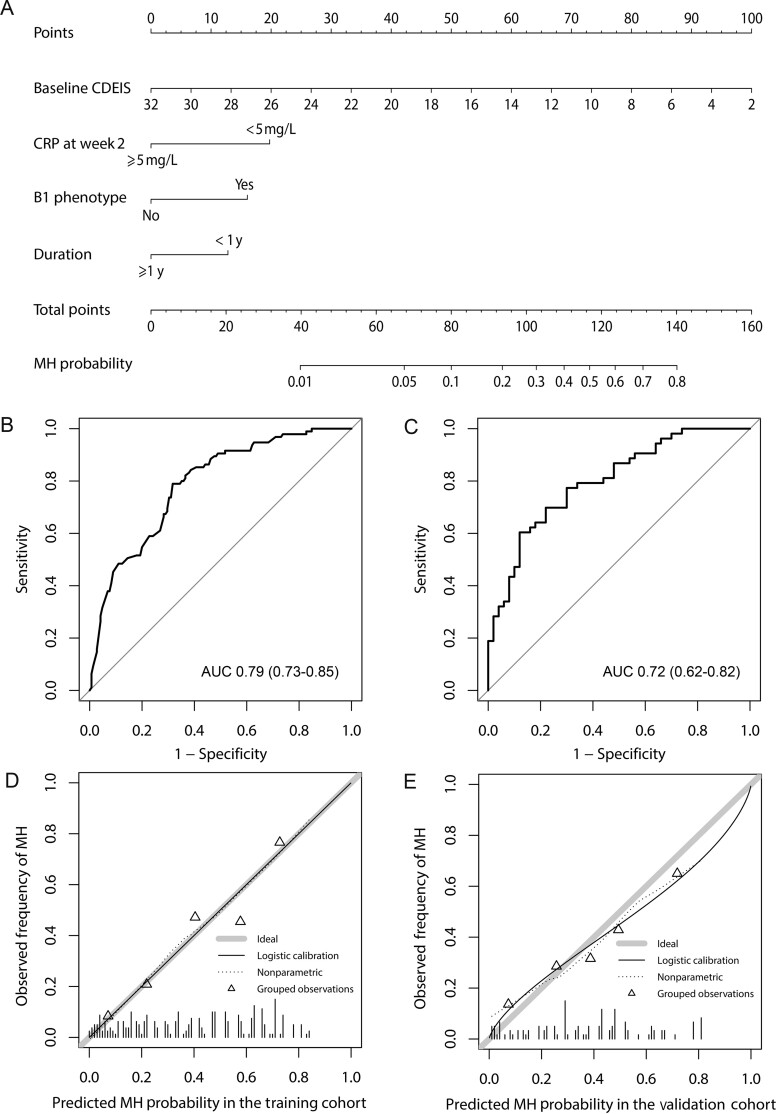
The display and evaluation of the model for mucosal healing (MH) responding to infliximab in patients with Crohn’s disease. (A) The nomogram to predict the probability of MH. Receiver-operating characteristic (ROC) curves for the MH nomogram in the training cohort (B) and validation cohort (C). Plots (D) and (E) show the calibration curves of the training and validation cohorts, respectively. AUC, area under ROC curve; B1 non-stricturing, non-penetrating behavior; CDEIS, Crohn's Disease Endoscopic Index of Severity; CRP, C-reactive protein.

Univariate and multivariate analyses were performed to identify significant factors for MH (Table 3). In univariate analyses, the B1 phenotype (*P* < 0.001) was positively associated with MH. In contrast, disease duration >1 year (*P* = 0.010), baseline CRP (*P* = 0.029), CRP ≥ 5 mg/L at week 2 (*P* < 0.001), and baseline CDEIS (*P* < 0.001) were negatively associated with MH. Four independent factors were identified by multivariate analyses, including duration of disease >1 year (OR 0.45; 95% CI, 0.24–0.85), B1 phenotype (OR 2.57; 95% CI, 1.25–5.31), CRP ≥ 5 mg/L at week 2 (OR 0.35; 95% CI, 0.14–0.87), and baseline CDEIS (OR 0.82; 95% CI, 0.76–0.89). The nomogram for MH is shown in Figure 4A.

**Table 3.** Univariate and multivariate logistic analyses for mucosal healing in 240 patients with Crohn’s disease of the training cohort

Variable	Univariate analysis		Multivariate analysis	
	*P* value	OR (95% CI)	*P* value	OR (95% CI)
Age (1-year increase)	0.132	0.98 (0.95–1.01)		
Male sex	0.269	1.40 (0.77–2.54)		
BMI ≥ 18.5 kg/m^2^	0.055	1.67 (0.99–2.81)		
Smoking	0.880	1.10 (0.34–3.56)		
Duration of disease > 1 year	0.010	0.50 (0.29–0.85)	0.013	0.45 (0.24–0.85)
Disease location				
L1: Ileal		Reference		
L2: Colonic	0.268	0.48 (0.13–1.77)		
L3: Ileocolonic	0.522	0.73 (0.29–1.89)		
Upper-tract involvement	0.058	1.77 (0.98–3.19)		
B1 phenotype	<0.001	3.02 (1.60–5.68)	0.011	2.57 (1.25–5.31)
Perianal disease	0.354	1.29 (0.75–2.20)		
Presence of extra-intestinal manifestations	0.894	1.04 (0.56–1.95)		
Prior surgery	0.176	1.44 (0.85–2.45)		
Baseline CRP (1-mg/L increase)	0.029	0.99 (0.98–1.00)	0.296	0.99 (0.98–1.06)
CRP ≥ 5 mg/L at week 2	<0.001	0.20 (0.09–0.46)	0.023	0.35 (0.14–0.87)
Baseline albumin (1-g/L increase)	0.080	1.04 (0.99–1.10)		
Baseline CDEIS (1-unit increase)	<0.001	0.82 (0.76–0.88)	< 0.001	0.82 (0.76–0.89)
Concomitant therapy with AZA/6-MP	0.180	1.43 (0.85–2.40)		
Concomitant therapy with corticosteroids	0.130	0.45 (0.16–1.27)		

6-MP, 6-mercaptopurine; AZA, azathioprine; B1, non-stricturing, non-penetrating behavior; BMI, body mass index; CI, confidence interval; CDEIS, Crohn's Disease Endoscopic Index of Severity; CRP, C-reactive protein; OR, odds ratio.

#### Performance of the nomogram

AUCs were 0.79 (95% CI, 0.73–0.85) and 0.72 (95% CI, 0.62–0.82) in the training cohort and validation cohort, respectively (Figure 4B and C). The *P*-values of the Hosmer-Lemeshow goodness-of-fit test were 0.241 and 0.346 in the training and validation cohorts, respectively. The calibration curves showed notable agreement between predicted MH probability and observed MH rate (Figure 4D and E).

#### Comparison of the nomogram with single factors

The discriminatory ability of the nomogram was significantly higher than that of disease duration, B1 phenotype, CRP at week 2, or baseline CDEIS in the combined training and validation cohorts (all *P* < 0.05; Figure 2B).

## Discussion

The differential responses of CD patients to IFX treatment present an avenue by which precision medicine, in the form of individualized treatment strategies, can be used to optimize CD therapies [13]. Treatment with Vedolizumab, another biologic CD therapeutic, is already indicated by a scoring system derived from clinical-trial data, which has been validated on real-world data [23]. However, IFX, as the first biologic approved for CD, still lacks a convincing and practical clinical prediction tool. IFX remains the predominant biologic treatment for CD in China, so a reliable tool for predicting IFX response is urgently needed. Previously, we reported that serum interleukin 9 levels were predictive of IFX clinical efficacy in CD patients at our center [24]. To obtain a more widely applicable predictive model, we performed this multicenter study and constructed two nomograms to predict PNR and MH in response to IFX treatment, which represented the worst and best outcomes, respectively. Both nomograms had a notable discriminatory ability in our multicenter cohort. Our study showed that PNR occurred in 25% of cases, which is consistent with previous studies [13], and the MH rate was ∼38%, which is similar to that in other studies (29%–45%) [25].

As a biomarker for inflammation, CRP has been shown to be associated with disease activity in CD patients [26] and played an important role in our nomograms. To summarize, CRP at week 2 were negatively related to response to IFX in our study. While several studies have illustrated that CRP at week 14 is a biomarker of clinical response to IFX in CD patients [27–29], we found that early normalization of CRP at week 2 was also indicative of IFX response. A similar result for the predictive capacity of CRP at week 2 has been previously reported in ulcerative colitis patients [30]. As for baseline CRP, there is no consensus on whether elevated CRP is related to response to IFX or how it affects the outcome [31]. In this study, we found that higher baseline CRP levels were negatively associated with response in univariate analysis, which is consistent with one previous report [27] but contrary to other studies [28, 29, 32]. Despite significance in univariate analysis, baseline CRP was not influential enough to enter the final model. Thus, physicians should not make decisions according to baseline CRP.

Low BMI may also predict disease-course severity [33]. We found that low BMI (BMI < 18.5 kg/m^2^) was negatively associated with IFX response, similarly to previously reported findings [19]. There have been numerous studies about the association between obesity and loss of response [34, 35], but investigations into the relationships between underweight and PNR merit further investigation. Since our patients had a lower BMI (median, 18.3 kg/m^2^; IQR, 15.9–20.0 kg/m^2^) than Western patients with CD [34, 35], we only explored the influence of underweight.

Other clinical factors that we identified have also been linked to response to IFX in previous studies. It has been reported that younger patients are more likely to respond to IFX than older patients [19, 36]. We also found that the probability of PNR elevated with increasing age, although the mechanism for this has not been fully elucidated. Some reports have suggested that patients with shorter disease duration have a higher chance of responding to IFX [37, 38]. We also found similar relationship between disease duration and response in this study. Consistently with previous studies [39, 40] and generally held beliefs, stenosing or fistulizing phenotypes were associated with worse clinical outcomes in our study. Additionally, studies about the relationship between disease severity and response to IFX are insufficient and remain controversial [31]. We found that more severe disease is not related to PNR but is less likely to achieve MH.

There are several advantages to the methodology and outcomes of our study compared to those of previous studies. First, our study made explicit comparisons between predictions of IFX response made by nomograms, by single factors included in the nomograms, and a previously published matrix model [19]. The nomograms developed in our study provided more accurate predictions than any single factors and the existing matrix model. Second, as our multicenter study included data from three geographically distinct IBD centers in China, comprising the southern, eastern, and northern parts of China, our results can be considered as representative of the patient population in China. Third, all data included in our study were routine clinical data, requiring no extra physical examinations or genetic characterization of patients, making the nomograms that we have developed both practical and economical for physicians, especially those in developing countries.

However, there are some limitations to our study. First, some clinical data, such as IFX levels and anti-IFX antibodies, were incomplete and not explored, while only half of the patients had combination therapy of azathioprine or 6-mercaptopurine. Second, > 60% of patients had perianal disease but only luminal disease outcomes were assessed for endpoints due to lack of a detailed record of perianal disease. Third, as patients were included with baseline CDEIS > 3, those with disease limited to the upper gastrointestinal tract and a substantial number of patients with lesions limited to the terminal ileum were excluded from this study, potentially biasing our cohort and limiting the scope of patients to which our nomograms are applicable. However, 70% of CD patients have disease located in the ileocolon or colon [41], so the nomograms we developed are still useful for the majority of CD patients. Moreover, since CDEIS was used as the definition of outcome, lesions in the upper gastrointestinal tract were not evaluated. Furthermore, the nomograms developed require prospective and external validation before they can be widely adopted.

In conclusion, our proposed nomograms provide accurate predictions of IFX-related PNR and MH in CD patients. To the best of our knowledge, this is the first nomogram to be developed from a multicenter cohort to predict response to IFX in CD patients. Using our nomograms to predict IFX response could reduce the time needed to identify effective therapeutic approaches for CD patients, saving costs and reducing patient harm.

## Authors’ contributions

Guarantor of the article: S.H.Z. S.H.Z., Q.C., and M.H.C. designed the study. X.Q.Y., J.C., Q.Y., X.C.C., M.X.R., Y.C., H.C., and Y.M.L. collected the data. X.Q.Y., J.C., B.L.C., Y.H., and Z.R.Z. analysed the data. S.H.Z. and X.Q.Y. wrote and revised the manuscript. All authors read and approved the final manuscript.

## Funding

This study was supported by grants from the National Natural Science Foundation of China (#81870374, #81670498), Guangdong Science and Technology (#2017A030306021), Science and Technology Innovation Young Talents of Guangdong Special Support Plan (#2016TQ03R296), and the Fundamental Research Funds for the Central Universities (#19ykzd11).
